# Federal vs. State Quarantine

**Published:** 1907-01

**Authors:** 


### EDITORIAL DEPARTMENT.

### FEDERAL VS. STATE QUARANTINE.

The Federal Quarantine Bill passed by the last Congress provides for the taking over by the United States Public Health and Marine Hospital Service, the duty and expense of international quarantine, and the purchase by the Federal government of the quarantine buildings and equipment owned by the State at a price to be agreed upon. In order to perfect the act of Congress and make it effective, the Legislature of each Southern quarantine State must, by special act, ratify and approve of such transfer, and it will then remain only to make deeds to the property and give possession. That is a mere detail.

It becomes now the duty of the Thirtieth Legislature just assembled, to pass such an act, to give effect to the Federal law in Texas. A bill for that purpose should be introduced at an early day, and no doubt will be.

It would seem that no argument should be necessary in support of such a proposition. Texas being a frontier State, pays for the protection of all that lies behind her, from epidemic invasion, which is neither just nor right. Moreover, her sanitary cordon on the Mexican border and on the gulf coast, for which some $50,000 a year is paid, is a superfluity, in as much as it is duplicated by the Federal government, anyhow, and thus a double line, one State and one Federal, is maintained to guard the State against—what? yellow fever! and that, too, to the neglect of internal sanitation to protect the people from indigenous diseases that destroy a hundred times more lives than yellow fever, and compared to which yellow fever is a minor danger: e. g.: according to figures compiled from the U. S. Census Reports by Reed, Carroll, and Agramonte, from 1793 to 1900 the average number of deaths from yellow fever *per year* was 1000, while the U. S. Census Reports for 1890 and 1900 show the number of deaths from consumption to be 150,000 a year, or one hundred and fifty times greater than from yellow fever: that whereas one thousand die of yellow fever in *a year,* one thousand die of consumption every *two and a half days!* Four hundred and eleven a day, or one every three minutes, night and day.

And yet the spread of consumption is preventable.

And yet nothing, practically nothing, is being done to prevent it!

It is true, some cars are "disinfected" after a fashion. Public buildings are not, although the law stipulates they should be.

The money saved to the State by letting Uncle Sam do the gulf and border quarantining would go a long way in enforcing sanitary measures for the prevention of diseases other than imported infection; for instance, it would make effective the Vital Statistics Act (Twenty-ninth Legislature) inoperative for want of $2000. It would make effective the pure food law, enacted in 1885, but a dead-letter for want of a small appropriation. It would make effective the disinfecting of cars, hotels and public buildings; the prevention of water pollution that breeds typhoid fever, *et cetera, et cetera.*

The whole medical profession of Texas favors the transfer of quarantine to the U. S. government.

I reproduce from a late Galveston paper the resolution adopted by the South Texas Medical Association in recent session in Houston, and add my endorsement of same:

THE RESOLUTION.

WHEREAS, The national quarantine law provides for the immediate establishment of quarantine stations at all gulf ports, regardless of existing State stations;

WHEREAS, The problem transcends a State duty, and being national, belongs to Federal supervision; and,

WHEREAS, One of the avowed policies of our American medical and the State medical associations, voicing the sentiment of the most intelligent men of our profession, was the confidence in the ability and experience of the United States public health and marine hospital service; and,

WHEREAS, Were our State government to maintain an independent quarantine system side by side with these uniform quarantine stations, it would be a calamity in that it would work a double hardship on freight and passenger traffic, and which, in our opinion, is unwarranted and an unnecessary expense to our State when maintained simply as a check on one of the most efficient and most thoroughly equipped quarantine systems on the face of the globe; and,

WHEREAS, Constant demands are being made by the profession, the press and the public for more consideration of internal health affairs, such as a State chemist, an inspection service for pure food and drugs, a prevention of pollution of water supply of our cities, and proper care of our dependent consumptives, lepers, etc.; therefore be it

*Resolved*, That the South Texas District Medical Association, believing that we, being most vitally interested by virtue of our being contiguous to the coast, and more amenable to yellow fever epidemics, heartily indorse the policies as outlined by the national quarantine law and urge our various committees on public health and legislation to take up this matter with their respective State Senators and members of the Legislature, and admonish them to use their vote and influence towards the consummation of the above ideas, i. e., a sale of State stations and equipment to the National government, whereby we will get the same, or better, protection as at present afforded, without expense to our State government; and be it further

*Resolved*, That our representatives be requested to provide for the establishment of a State board of health and allow the present annual appropriation for maintenance of the quarantine service to be used by the board for the purpose of safeguarding the public health by dealing intelligently with problems of more vital interest at this time than yellow fever epidemics.

